# Development and Implementation of a Quick Response (QR) Code System to Streamline the Process for Fellows’ Evaluation in the Pediatric Intensive Care Unit (PICU) and the Neonatal Intensive Care Unit (NICU) at a Large Academic Center

**DOI:** 10.7759/cureus.47462

**Published:** 2023-10-22

**Authors:** Sara K Kane, Elizabeth A Wetzel, Jason Z Niehaus, Samer Abu-Sultaneh, Andrew Beardsly, Melissa Bales, Deb Parsons, Courtney M Rowan

**Affiliations:** 1 Pediatrics/Neonatology, Indiana University School of Medicine, Indianapolis, USA; 2 Pediatrics/Critical Care Medicine, Indiana University School of Medicine, Indianapolis, USA; 3 Pediatrics, Indiana University School of Medicine, Indianapolis, USA

**Keywords:** medical residencies, educational technology, graduate medical education, evaluations, feedback (learning)

## Abstract

Background/objective: Useful feedback and evaluation are critical to a medical trainee’s development. While most academic physicians understand that giving feedback to learners is essential, many do not consider the components of feedback to be truly useful, and there are barriers to implementation. We sought to use a quick reader (QR) system to solicit feedback for trainees in two pediatric subspecialties (pediatric critical care and neonatal-perinatal medicine) at one institution to increase the quality and quantity of feedback received.

Methods: New valuations were modified from the existing evaluations and imported into online systems with QR code capability. Each fellow was given a QR code linking to evaluations and encouraged to solicit feedback and evaluations in a variety of clinical settings and scenarios. Evaluation numbers and quality of evaluations were assessed and compared both pre- and post-intervention.

Results: There were increases in the number of evaluations completed for both the pediatric critical care fellows and the neonatal-perinatal medicine fellows. There was no overall change in the quality of written evaluations received. Satisfaction with the evaluation system improved for both faculty and fellows of both training programs.

Conclusion: In our critical care units, we were successfully able to implement a QR code-driven evaluation for our fellows that improved access for the faculty and offered the ability of the learner to solicit evaluations, without compromising the number or quality of evaluations.

What’s new: Quick reader (QR) codes can be used by learners to solicit evaluations and feedback from faculty. They can increase the quantity of written evaluations received without affecting their quality.

## Introduction

Useful feedback and evaluation are critical to a medical trainee’s development. The literature repeatedly emphasizes the importance of feedback in medical training [1-3]. The Accreditation Council for Graduate Medical Education (ACGME) requires that trainees be evaluated regularly [4]. Opportunities for independent educational growth must be assisted with timely and specific feedback and evaluation [5-7]. Additionally, feedback should be received in multiple ways including both verbal feedback and feedback via written evaluations [6,8].

While most academic physicians understand that giving feedback to learners is essential, many do not consider the components of feedback to be truly useful. Feedback should be timely, objective, and specific; focus on behaviors that the learner can change; and offer concrete examples of what was right or wrong with their performance [8-11]. Feedback should also be solicited by the learner [9,12].

Although educators understand the value of feedback and evaluation, there are barriers that exist to implementation. When considering written evaluations, common barriers include time, accessibility, and lack of focus on the evaluation [13]. Our previous evaluations were long, had several unfocused questions, required faculty to sit at their desks to complete, and often came weeks or months after encounters with fellows. We hypothesized that by making the evaluation system more accessible, empowering our fellows to solicit evaluations, and focusing on the evaluation content, we would increase the quality and quantity of written feedback received.

## Materials and methods

Setting and participants 

This evaluation method was initially designed and implemented for use in the pediatric critical care fellowship and then adapted to be used in the neonatal-perinatal medicine fellowship at Indiana University School of Medicine, Riley Hospital for Children. The studies were reviewed and exempted by the Indiana University Institutional Review Board.

The pediatric critical care fellowship at Indiana University admits two fellows each year for a three-year fellowship. Fellows do 17 months of clinical service, which consists of rotations in the general pediatric intensive care unit (PICU) and cardiac intensive care unit (CVICU). They also do regularly scheduled night and weekend calls throughout all months of their fellowship. The number of faculty during the study timeframe ranged from 15 to 18. Attending physicians cover the units in one-week blocks and also supervise during night calls.

The neonatal-perinatal medicine fellowship at Indiana University admits three fellows each year for a three-year fellowship. Clinical service time for fellows ranges from 12 to 20 months depending on the fellow’s individual pathway and includes time rotating through a level IV neonatal intensive care unit (NICU), a level III NICU with a high-risk delivery service, a level II NICU, the CVICU, and a perinatal consult service. They also do regularly scheduled night and weekend calls throughout all months of their fellowship. There were 32 faculty members during the study timeframe. Attending physicians cover the units in two- to three-week blocks and supervise during night calls.

Evaluation design and construction

On July 1, 2014, the PICU implemented a new evaluation system for the critical care fellows as a response to the new ACGME milestones [14]. The milestones were split into five evaluations: clinical week of service, night call, procedural, leadership skills/teamwork/professionalism, and patient/family conversation. A blank evaluation was also added should the faculty member desire to share more information or give feedback for an event they felt did not fit any of the categories (Table 1).

**Table 1:** Pediatric Critical Care Fellowship Evaluations *All evaluations were now linked to milestones. **360 evaluations include evaluations by any person who is not a pediatric critical care attending. Examples include bedside nursing, respiratory therapist, pharmacist, and advanced practice providers. These are always available on the unit to be completed. QR: quick response.

Evaluation system prior to QR code-linked evaluation		
Comprehensive biannual evaluations by attendings included the following: Clinical and communication skills, procedural skills, medical knowledge, presentation skills, annual 360 evaluation (i.e., nurse, respiratory therapist, pharmacist)		
QR Code-Linked Evaluation System*		
Clinical/Rotational	Procedure	Education/Conference
Patient care/service clinical week of service, Night call communication, Professionalism/teamwork/teaching, Family/Patient communication	Intubation arterial line, Central line, Thoracentesis, Chest tube placement, Resuscitation other	M&M Journal Club Research updates
		Other evaluations
		Readily available 360** Blank evaluation Self-evaluation

We then designed the system to operate with a quick response code (QR code) functionality. A QR code is a square barcode that is linked to a website. This barcode can be scanned with a smartphone or tablet. Scanning the code launches the web-based evaluation. A unique QR code was created for each fellow’s evaluation using a free online QR generator (www.qrstuff.com). Each learner carries their own code on their physician badge and is empowered to solicit feedback from their supervising physician at the time of the event; for example, immediately after a procedure is performed. The faculty member still had independent access to each fellow’s QR code, enabling the faculty member to grant unsolicited feedback. A master sheet with all QR codes was sent out to all faculty members to be saved on office desktop computers. It was placed around the office space and working areas in the PICU and CVICU for easy access. When the QR code was implemented in the 2014-2015 academic year, a faculty development session instructed faculty and fellows on how this system could be utilized.

A system using the same QR technology was then implemented with the neonatal-perinatal medicine fellows in January 2019. The neonatal milestones were reconfigured from seven different evaluation types into 27 different evaluations due to the realization that many clinical encounters were not being evaluated in the current system (Table 2).

**Table 2:** Neonatal-Perinatal Medicine Fellowship Evaluations *Previous evaluations were linked to milestones. **Previous evaluations were NOT linked to milestones. All others are novel evaluations. IU: Indiana University, NP: nurse practitioner, NNP: neonatal nurse practitioner, PA: physician assistant.

Clinical/Rotational	Procedure	Teaching/Education/Conference/Sim
Patient care/service, Admission changeover, Code blue in the unit, Daytime rounds, Delivery room resuscitation, End of rotation*, Friday feedback**, Night/weekend call*, Night rounds, Transport call, Communication/consults, Family communication*, IU talk communication, Inpatient consult (detailed), Inpatient consult (general)	General procedure evaluation for abdominal paracentesis, chest tube placement, exchange transfusion, intraosseous line, intubation, lumbar puncture, arterial line, pericardiocentesis, thoracentesis, umbilical line, other	Clinical talk*, Consult guideline paper M&M Conference Monday morning conference, Research talk**, Simulation*, Teaching session for NP, resident, or medical student
		Other evaluations, Kudos/comments, Fellow to fellow evaluation**, 360 evaluation*, NNP/PA 360*

The evaluations were placed under the categories of patient care/service, communication/consults, procedures, teaching/education/simulation, and others. In addition, there were blank evaluations that could be completed by faculty, advanced practice providers (APPs), nurses, respiratory therapists, and pediatric residents. The system was designed to work with QR code functionality, with the codes being worn on the fellows’ badges. Similar to critical care medicine, the faculty member had independent access to each fellow’s QR code; master sheets with all the fellows' QR codes were sent out to all faculty members to be saved on office desktop computers and were placed around the office space, working areas of the NICU, and available at all conferences. For both the PICU and NICU evaluations, the overall content of the evaluations was unchanged between the systems.

Quality assurance measure

For each group of fellows, six months of evaluations was pulled from the pre-QR code implementation system and compared to six months of evaluations from the immediate post-QR code implementation period. All evaluations were included in these time frames. The pre- and post-implementation word counts were evaluated with the speculation that longer comments likely yield more informative feedback. In addition, comments from the evaluations were extracted. These comments were mixed together on a spreadsheet and were reviewed by three physician educators for three pre-selected components of feedback. The reviewers were blind to which comments were pre- or post-QR code system. They evaluated the comments obtained from these evaluations for important elements of feedback [8-10]. The elements of feedback investigated were as follows: (1) Was the comment specific and descriptive? (2) Did it identify a correctable behavior? (3) Did it include a concrete example? Comments were scored independently. Differences in the scores of the three reviewers were discussed and adjudicated.

Data collected

For the pilot study of pediatric critical care fellows, the total number of evaluations was compared for the academic year (2013-2014) pre-QR code implementation with the two academic years (2014-2016) post-QR code. During the pre-QR code period, there were five fellows. After the post-QR code period, there were six fellows (four of whom were part of the pre-QR code cohort). The types of evaluations completed were also collected. Finally, the pediatric critical care faculty group was surveyed to determine faculty satisfaction with the new QR system.

For the neonatal fellows, the total number of evaluations per fellow was compared for the previous six months prior to implementation (July-December 2018) with the first six months post-implementation (January-June 2019). This time frame was selected to include the same seven fellows for both the pre-implementation and post-implementation phases. The neonatal-perinatal medicine faculty and fellows were surveyed to determine satisfaction with the new QR system.

Statistical analysis

The categorical variables are displayed as frequencies and percentages. These were compared with chi-squared and Fisher's exact analysis. Continuous variables were reported as means and standard deviations and were compared using a paired t-test. Medians and interquartile ranges were compared using the Mann-Whitney U test. Statistical Package of Social Science (SPSS) for Windows, Version 22 (SPSS Inc., Chicago IL) and Microsoft Office Excel (Microsoft, Corporation, Redmond WA) were used for analysis.

## Results

Number of evaluations

**Figure 1:**
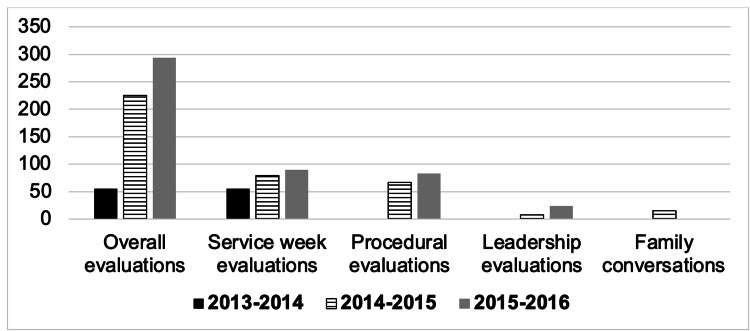
PICU Evaluation Counts PICU: pediatric intensive care unit.

After implementing the QR code evaluation system for pediatric critical care fellows, there was a drastic increase in the number of evaluations: 55 (average of 11 per fellow) in the pre-QR period compared to 225 (average of 37.5 per fellow, p≤0.001) and 294 (average of 49 per fellow, p≤0.001), in the two academic years post-QR implementation (Figure 1).

The service week filled evaluations increased from 55 to 79 (p=0.001) and then 90 (p≤0.001) for the 2013-2014, 2014-2015, and 2015-2016 academic years, respectively. During the QR period, there was also an improvement in the number of procedural evaluations (67 to 83), leadership evaluations (eight to 24), family conversations (15 to 27), and blank evaluations (one to two) from 2014 to 2015 to 2015 to 2016 academic years, respectively.

After implementing the QR evaluation system for neonatal-perinatal fellows, there was also an increase in the number of evaluations. In the six months prior to implementation, there were 75 total evaluations compared to 276 evaluations in the six months following. Because the evaluation categories were different, we were unable to analyze to see an increase in specific subtypes of evaluation categories.

Quality assurance

In the pediatric critical care fellowship, 83 (50 pre-QR period and 33 post-QR period) comments were evaluated for feedback quality. As seen in Table 3, the median word count per evaluation did not change between the periods. There was also no change in the quality of the feedback provided in the evaluation comments, including whether the comments were descriptive, whether a correctable behavior was identified, or whether a concrete example was given.

**Table 3:** Critical Care Fellowship Pre-QR Period and Post-QR Period Description of Comments QR: quick response, IQR: interquartile range.

Pediatric Critical Care Fellowship		
Pre-QR Period	Post-QR Period	p-value
Overall comments: 50	Overall comments: 33	
Median word count: 20.5 (IQR: 11.75-32.0)	Median word count: 21 (IQR: 11.0-26.5)	0.52
Descriptive comments: 86%	Descriptive comments: 87.9%	0.81
Correctible behavior identified: 94%	Correctible behavior identified: 84.8%	0.17
Concrete example given: 40%	Concrete example given: 54.5%	0.19

For neonatal fellows, 252 comments (53 pre-QR period and 199 post-QR period) were evaluated for feedback quality. As seen in Table 4, the median word count per evaluation statistically significantly decreased between the periods. There was no change in comments being descriptive or a concrete example being given, but correctable behaviors were identified more often in the pre-QR comments than in the post-QR comments.

**Table 4:** Neonatal-Perinatal Fellowship Pre-QR Period and Post-QR Period Description of Comments QR: quick response, IQR: interquartile range.

Neonatal-Perinatal Medicine Fellowship		
Pre-QR Period	Post-QR Period	p-value
Overall comments: 53	Overall comments: 199	
Median word count: 70.8 (IQR: 30-111.6)	Median word count: 35.4 (IQR: 21.2-49.6)	0.01
Descriptive comments: 100%	Descriptive comments: 89.9%	0.06
Correctible behavior identified: 54.7%	Correctible behavior identified: 36.7%	0.02
Concrete example given: 60.4%	Concrete example given: 52.3%	0.29

Faculty satisfaction

All of the faculty from pediatric critical care medicine agreed that the QR code evaluations were easy to access. Ninety percent of the faculty endorsed completing the evaluations within one week of working with the fellows. The faculty felt that 50% of the time the fellow was approaching them for evaluations at the end of a procedure, conversation, or week of service and 40% of the faculty reported being approached approximately once a month. Finally, 90% of the faculty preferred the new QR drive evaluation system compared to the previous version.

The majority (77%) of faculty from neonatal-perinatal medicine felt that the new system was easier to evaluate, and the same number felt that the new system took less time to complete an evaluation. Ninety-five percent of faculty felt as though the evaluations were just as good or better than the old system. Finally, 83% of faculty preferred the new system of evaluations.

Fellow satisfaction

Upon surveying the fellows from neonatal-perinatal medicine, six of the seven fellows (85.7%) felt that the QR code evaluations gave an accurate assessment of their clinical abilities, and all felt that the evaluations were just as good as or better than the old system. All fellows preferred the new system of evaluations. Fellows also reported that the QR code system provided them with more frequent face-to-face feedback. The main downside reported by fellows was that feedback was more often prompted by the fellows than by the faculty (71.4%).

## Discussion

In our study, two different pediatric fellowship training programs were able to successfully implement a QR code-driven evaluation system. While evaluation details between the divisions differed, both the pediatric critical care fellowship and neonatal-perinatal medicine fellowship at a large quaternary care center were able to increase the number of evaluations without compromising the quality of the feedback received. Furthermore, the faculty responsible for completing the evaluations were satisfied with the new system and overwhelmingly preferred the traditional, desktop computer-driven system.

There are several reasons that this implementation was successful. One of the reasons, which is unique to this system, is the ability of the learner to solicit feedback directly from the supervisor. Our learners all carried their own evaluation QR codes on their identification badges. This empowered the learner to request on-the-spot feedback, by offering their QR code to the supervisor at the point of contact of the encounter. This reminded the physician in charge to fill out an evaluation, prompted oral feedback to the learner, and provided the learner with a sense of responsibility and ownership for their own education and clinical growth. In a busy clinical environment, it is the responsibility of the learner and teacher to set time for feedback, ideally within a timely manner [6,9]. Both parties must be invested in realizing its importance. It must be both solicited and freely given to be successful.

The QR code evaluation system also likely resulted in an increased number of evaluations due to ease of access. Having the system readily available on multiple technological interfaces such as the computer, tablet, or smartphone made the evaluations more accessible. It is important to adapt educational methods to keep pace with our ever-changing social environment [15]. The QR code evaluation system is a great example of this. Smartphones are ubiquitous. In 2016, a survey of medical students and physicians revealed that 94% owned a smartphone, and 83% used them daily in clinical practice [16]. Clearly, the use of mobile technology is extremely common in the medical field and it is being applied to an increasing amount of education endeavors. By having the option to complete evaluations on the phone or tablet as opposed to being confined to a computer, faculty members can complete the evaluations in a more timely fashion and more directly to the experience observed.

There was some difference in quality analysis between the two groups of fellows. For the pediatric critical care fellows, despite many evaluations being completed by smartphone, the quality of evaluations was not affected. Important aspects of feedback such as the comments being descriptive, identifying a correctable behavior, and providing a concrete example were not different from the implementation of the QR system. While not reaching statistical significance, we did notice there was a large increase, from 40% in the pre-QR period to almost 55% in the post-QR period, in the comments identifying a concrete example. For the neonatal-perinatal fellows, the median word count decreased after the implementation, the comments were scored as less descriptive and less frequently identifying a correctable behavior. We believe this difference is due to the fact that many new specific evaluations were added in the new system, so faculty were completing a greater number of shorter evaluations in lieu of a few longer evaluations and the evaluations were more specific based on the activity being evaluated. We hypothesized that faculty find it easier to write shorter comments when using a cell phone vs. typing an evaluation on a computer. We also hypothesize that more face-to-face feedback was occurring in place of writing longer, detailed comments. With the improved accessibility and availability of the learner to solicit feedback on the spot, there may be more timely, insightful feedback provided. Although the neonatal-perinatal fellows felt the learner-driven facilitation of feedback was a downside, the best feedback is that which is solicited by the learner [9,12].

Our study has some limitations. First, the ever-changing fellow population and change in the size of our faculty group over time may affect the number of evaluations. We hope that this limitation is minimized by the fact that our fellowship size has not changed over time, nor has the amount of clinical time done by the fellow changed. In the NICU evaluations, while the questions in the evaluations remained the same, larger evaluations were broken up into smaller, shorter evaluations to allow for ease of completion in more real-time scenarios. This could have impacted the overall increase in number of evaluations completed. Another limitation is that we do not have data on where the faculty are completing the evaluations (i.e., on the desktop computer, smartphone, or tablet). Regardless of where the evaluations are being done, it is a unique aspect of the QR code to offer a variety of mechanisms to complete evaluations that will fit the style of the individual teacher the best.

## Conclusions

In our critical care units, we were successfully able to implement a QR code-driven evaluation of our fellows that improved access for the faculty and offered the ability of the learner to solicit evaluations, without compromising the number or quality of evaluations. Faculty and fellows were both satisfied with the quality of feedback they received. It would be important moving forward to test this system in other learning environments and at other institutions to validate its effectiveness.
